# Finding the right balance: ASY1 recruits opposing regulators to finetune DMC1 function during meiosis

**DOI:** 10.1093/plcell/koag248

**Published:** 2026-08-17

**Authors:** Julie Robinson

**Affiliations:** Assistant Features Editor, The Plant Cell, American Society of Plant Biologists, United States; HudsonAlpha Institute for Biotechnology, Huntsville, AL 35806, United States

Meiosis is a tightly regulated event with many players that work in concert to ultimately generate haploid cells for sexual recombination. During this complex process, double-strand breaks (DSBs) are created to facilitate crossover between homologous chromosomes. DMC1 is the main recombinase responsible for homology search and subsequent DSB repair, without which formation of bivalents fails and fertility is drastically reduced (Couteau et al. 1999). ASY1 is a HORMA-domain-containing protein that plays an important role in homology search and strand invasion, and previous research has indicated that ASY1 regulates DMC1 loading onto chromosomes (Sanchez-Moran et al. 2007). However, the mechanism by which this is achieved has been unclear, especially since ASY1 does not seem to recruit DMC1 to chromosomes through influencing the formation of DSBs. In new work, **Cai and colleagues** (Cai et al. 2026) elucidate the molecular mechanisms by which ASY1 regulates DMC1 localization during early meiosis in *Arabidopsis thaliana* and *Brassica napus*.

In wild-type cells, DMC1 localizes onto chromosomes during early prophase I (leptotene) and disappears by mid prophase I (pachytene). In *asy1* mutants, DMC1 remains in the nucleus, but its association with DNA—visible via live-cell imaging and immunostaining—decreases (Fig. 1b), and it persists later in prophase I (diplotene). These observations suggest a role for ASY1 in loading DMC1 onto chromosomes and that timely removal/degradation of DMC1 depends on proper synapsis. However, yeast two-hybrid and BiFC assays both show that ASY1 and DMC1 do not physically interact. Cai and colleagues thus turned to SDS, a plant-specific meiotic cyclin-like protein that promotes DMC1 localization—in its absence, DMC1 localization to chromatin is lost (Fig. 1; De Muyt et al. 2009). Fluorescence imaging and immunostaining show a complete lack of SDS signal on chromosomes in *asy1* mutants, indicating that ASY1 plays a critical role in recruiting SDS. Furthermore, split-luciferase complementation and BiFC assays show a direct physical relationship between ASY1 and SDS.

**Figure 1:**
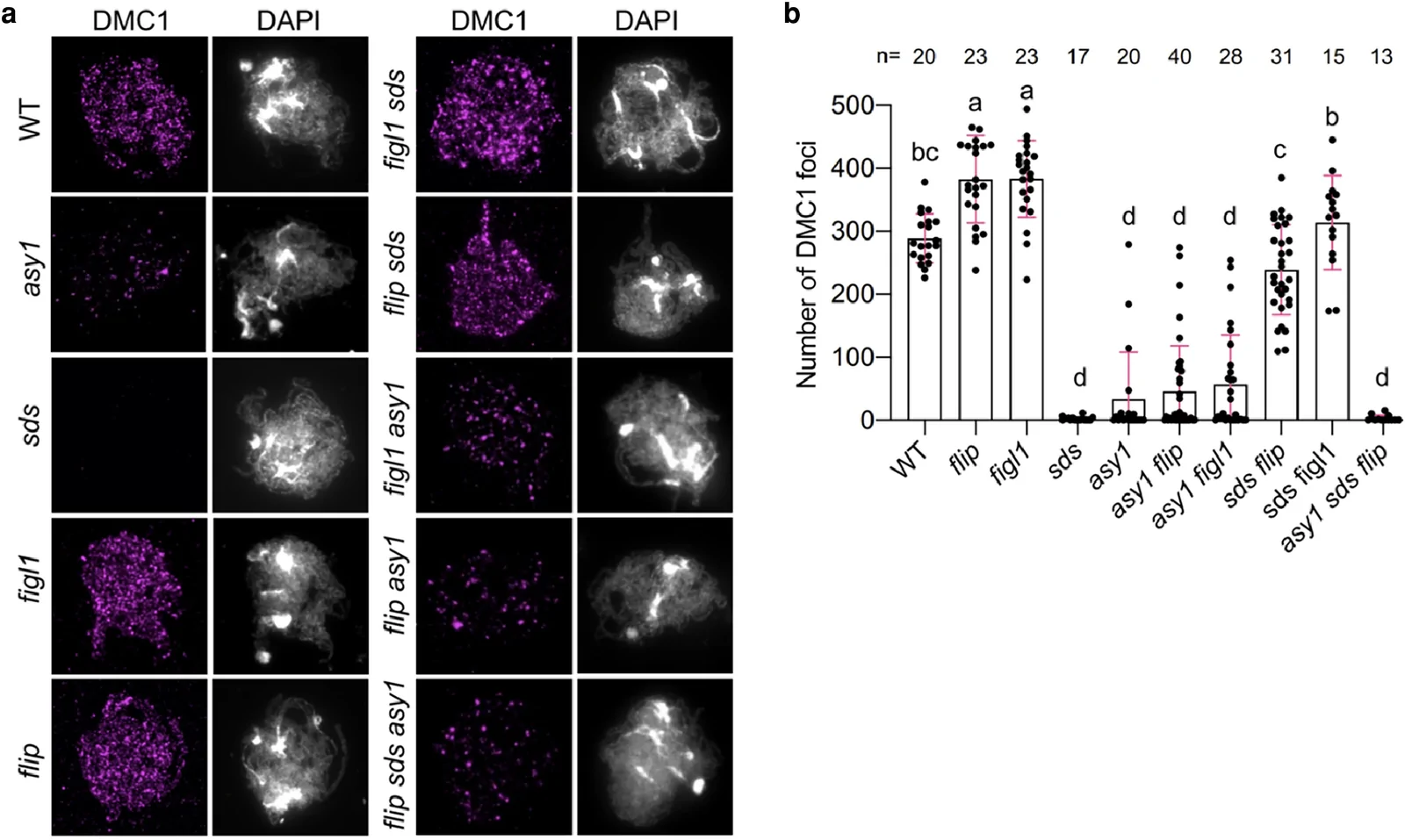
a) DMC1 immunostaining and DAPI staining in various *Arabidopsis thaliana* mutants during early prophase I. b) Quantification of DMC1 foci in (A). Adapted from Cai et al. (2026), Fig. 8.

In an elegant experiment, Cai and colleagues utilized a *zyp1* mutant, which is unable to clear ASY1 from chromosomes, to demonstrate that ASY1 promotes SDS recruitment to chromosomes. In *zyp1* mutants, SDS persists on chromosomes longer than in wild-type cells; however, in *zyp1 asy1* double mutants, SDS does not localize to chromosomes at all. Next, the researchers sought to understand the mechanism by which ASY1 regulates SDS recruitment and by extension that of DMC1. Deletion of a conformational regulatory element called the closure motif of ASY1 compromised SDS and DMC1 localization. Despite contradictory results regarding whether the physical interaction between SDS and this mutant ASY1 is lost or not, it still appears that the closure motif affects the conformation of ASY1 in such a way that influences its ability to interact properly with SDS along chromosomes.

Finally, because DMC1 localization is also known to be negatively regulated by the FIGL1-FLIP complex (Fernandes et al. 2018), the researchers performed an epistasis analysis to determine whether it acts upstream or downstream of ASY1. While mutating FIGL1 or FLIP in an *sds* mutant background partially rescues DMC1 chromosome localization and plant fertility, such mutation in an *asy1* mutant background does not (Fig. 1). This finding indicates that FIGL1-FLIP acts downstream of ASY1.

Considering their findings together, Cai and colleagues propose that ASY1 acts as a scaffold to create a chromatin environment conducive to appropriate DMC1 activity by recruiting opposing DMC1 regulators—the positive regular SDS and the negative regulator FIGL1-FLIP—to spatiotemporally fine-tune that activity. Future research in this area will investigate the manner in which SDS promotes loading of DMC1 onto chromosomes and the molecular nuances of the chromatin environment during prophase I that permit efficient recombination.

Recent related articles in *The Plant Cell*:

- Trujillo-Esquivel et al. (2026) functionally characterized the homologous recombination protein RAD51 in *Marchantia polymorpha*.
- Zhu et al. (2024) used crossover mapping to define a role of ATR kinase in crossover distribution in *Arabidopsis thaliana*.
- Chu et al. (2024) investigated the function of the meiotic regulator ASY3 in *Brassica napus*.

## Data Availability

No new data were generated or analyzed in support of this article.
